# Zoonotic *Abbreviata caucasica* in Wild Chimpanzees (*Pan troglodytes verus*) from Senegal

**DOI:** 10.3390/pathogens9070517

**Published:** 2020-06-27

**Authors:** Younes Laidoudi, Hacène Medkour, Maria Stefania Latrofa, Bernard Davoust, Georges Diatta, Cheikh Sokhna, Amanda Barciela, R. Adriana Hernandez-Aguilar, Didier Raoult, Domenico Otranto, Oleg Mediannikov

**Affiliations:** 1 IRD, AP-HM, Microbes, Evolution, Phylogeny and Infection (MEPHI), IHU Méditerranée Infection, Aix Marseille Univ, 19-21, Bd Jean Moulin, 13005 Marseille, France; younes.laidoudi@yahoo.com (Y.L.); hacenevet1990@yahoo.fr (H.M.); bernard.davoust@gmail.com (B.D.); didier.raoult@gmail.com (D.R.); 2 IHU Méditerranée Infection, 19-21, Bd Jean Moulin, 13005 Marseille, France; georges.diatta@ird.fr (G.D.); cheikh.sokhna@ird.fr (C.S.); 3 Department of Veterinary Medicine, University of Bari, 70010 Valenzano, Italy; maria.latrofa@uniba.it (M.S.L.); domenico.otranto@uniba.it (D.O.); 4 IRD, SSA, APHM, VITROME, IHU Méditerranée Infection, Aix-Marseille University, 19-21, Bd Jean Moulin, 13005 Marseille, France; 5 VITROME, IRD 257, Campus International UCAD-IRD, Hann, Dakar, Senegal; 6 Jane Goodall Institute Spain and Senegal, Dindefelo Biological Station, Dindefelo, Kedougou, Senegal; amanda.b@janegoodall.es (A.B.); r.a.hernandez-aguilar@ibv.uio.no (R.A.H.-A.); 7 Department of Social Psychology and Quantitative Psychology, Faculty of Psychology, University of Barcelona, Passeig de la Vall d’Hebron 171, 08035 Barcelona, Spain

**Keywords:** *Abbreviata caucasica*, *Physaloptera mordens*, *Pan troglodytes verus*, wild chimpanzees, nematode, zoonosis, Senegal

## Abstract

*Abbreviata caucasica* (syn. *Physaloptera mordens*) has been reported in human and various non-human primates including great apes. The identification of this nematode is seldom performed and relies on egg characterization at the coproscopy, in the absence of any molecular tool. Following the recovery of two adult females of *A. caucasica* from the feces of wild Senegalese chimpanzees, morphometric characteristics were reported and new data on the width of the esophagus (0.268–0.287 mm) and on the cuticle structure (0.70–0.122 mm) were provided. The molecular characterization of a set of mitochondrial (*cox*1, 16S rRNA, 12S rRNA) and nuclear (18S rRNA and ITS2) partial genes was performed. Our phylogenetic analysis indicates for the first time that *A. caucasica* is monophyletic with *Physaloptera* species. A novel molecular tool was developed for the routine diagnosis of *A. caucasica* and the surveillance of Nematoda infestations. An *A. caucasica*-specific qPCR targeting the 12S gene was assessed. The assay was able to detect up to 1.13 × 10^−3^ eggs/g of fecal matter irrespective of its consistency, with an efficiency of 101.8% and a perfect adjustment (R^2^ = 0.99). The infection rate by *A. caucasica* in the chimpanzee fecal samples was 52.08%. Only 6.19% of the environmental samples were positive for nematode DNA and any for *A. caucasica*. Our findings indicate the need for further studies to clarify the epidemiology, circulation, life cycle, and possible pathological effects of this infestation using the molecular tool herein developed.

## 1. Introduction

Physalopteriasis is caused by parasitic nematodes from the genus *Physaloptera* (Spirurida, Physalopteridae) [1], which has been distributed in Africa and the Middle East (i.e., Iran) since prehistoric times [2,3]. Following the first formal description of the *Physaloptera* genus (Rudolphi in 1819), *Physaloptera abbreviata* (Rudolphi, 1819) was designated as the type species [4]. Afterward, *Abbreviata* was defined as a distinct genus, based on the number, mode, and origin of the uteri, which constitute the main keys for genus differentiation [4]. Adult stages of this genus are found in the stomach of a variety of animals such as reptiles and mammals including humans [5].

*Abbreviata* (=*Physaloptera*) *caucasica* (Linstow 1902), is a gastrointestinal nematode of Simiiformes (Anthropoidea) members [6]. After its discovery in a Caucasian man, Linstow (1902) provided an incomplete description with some erroneous morphological details [7]. In 1926, Schulz gave the complete description of *A. caucasica* after re-examining the original specimens, establishing a close relationship of this species with *Physaloptera mordens* (Leiper, 1908) isolated from humans in Central Africa. The unique difference identified among specimens was the presence/absence of a series of small teeth between and exterior to the large teeth on the inner face of the lip of *A. caucasica* and *P. mordens*, respectively [8]. A few months later, Ortlepp re-examined *P. mordens* and confirmed that the small teeth were missing in the previous examinations [9], therefore synonymizing *P. mordens* (Leiper, 1908) and *A. caucasica* (Linstow, 1902). 

The *A. caucasica* infection has been reported in New and Old World monkeys [10], rhesus macaques (*Macaca mulatta*), baboons (*Papio* spp.), and great apes including both captive (*Pongo* spp.) and wild (*Pongo abelii*) [1,11]. It has also been reported in chimpanzees (*Pan troglodytes*) from Gombe (Tanzania) and Ngogo (Uganda) [12,13], although no adult specimens were examined. Furthermore, eggs of *Physaloptera* sp. have been reported in wild Senegalese chimpanzees [12,14]. Human and non-human primates probably constitute the natural host for *A. caucasica* [8]. The adult parasite looks like *Ascaris* sp. under the naked eye [15] and occurs in the digestive tract from the esophagus to the small intestine, where it can induce serious disease manifestations such as abdominal pain, anorexia, vomiting, and bloody diarrhea [2]. Records of the clinical signs in infected chimpanzees are lacking [6]. Nowadays, the detection of *A. caucasica* depends on the identification of eggs in the feces or in the detection of adult stages during post-mortem examination of the gastrointestinal tract of infested hosts [1]. The arthropod intermediate and/or paratenic hosts remain unknown though some experimental evidence indicates that *A. caucasica* could develop to the infective stage in *Blatella germanica* and in *Schistocerca gregaria* [5]. For other *Physaloptera* species, the intermediate arthropod host has been assessed such as *Tribolium confusum* [16], ground beetles, *Harpalus* sp. [17] as well as crickets, *Acheta assimilis*, and grasshoppers, *Melanoplus femurrubrum* [18,19].

Under natural conditions, *A. caucasica* may develop in arthropods and infestation probably occurs through ingestion of beetles, crickets, or other arthropods as well as paratenic hosts containing infective larvae [8,15]. However, the potential involvement of up to 28 paratenic and second intermediate hosts is suspected [6]. Anthelminthic drugs have been reported to be effective for the treatment of physalopteriasis in non-human primates [1]. However, their control should be reinforced by a molecular characterization to avoid the misleading conclusions about this parasite, sanitation and control of the potential paratenic or arthropod hosts as well as the surveillance of the infestation from the colon of non-human primates [1,16]. 

As part of the control of infectious and zoonotic diseases in the current chimpanzee population from the Dindefelo Community Natural Reserve in Senegal, we present here morphometric and phylogenetic findings to support the occurrence of *A. caucasica* in chimpanzees, providing a molecular characterization of a set of target mitochondrial (*cox*1, 16S rRNA, 12S rRNA) and nuclear (18S rRNA and ITS2) genes and morphological identification of adult specimens collected from feces of West African chimpanzees in Senegal. In addition, we developed a molecular test that could be used in a routine diagnostic laboratory instead of the labor-intensive coprological methods. The molecular test provides detection, egg quantification, and genetic characterization of *A. caucasica* from biological samples. We therefore applied this tool to a surveillance process and molecular xenomonitoring of *A. caucasica* from possible intermediate hosts and our current population of West African chimpanzees from Senegal. 

## 2. Results

### 2.1. Morphological Characteristics of Adult A. caucasica

**Figure 1:**
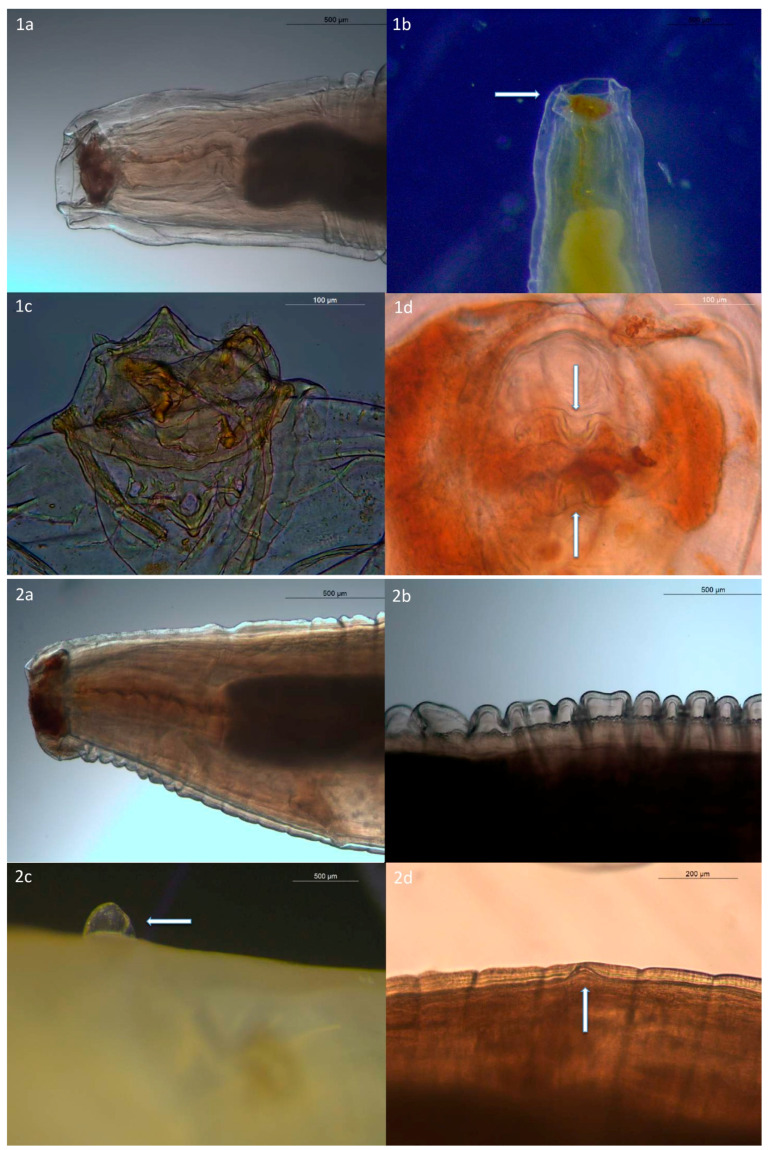

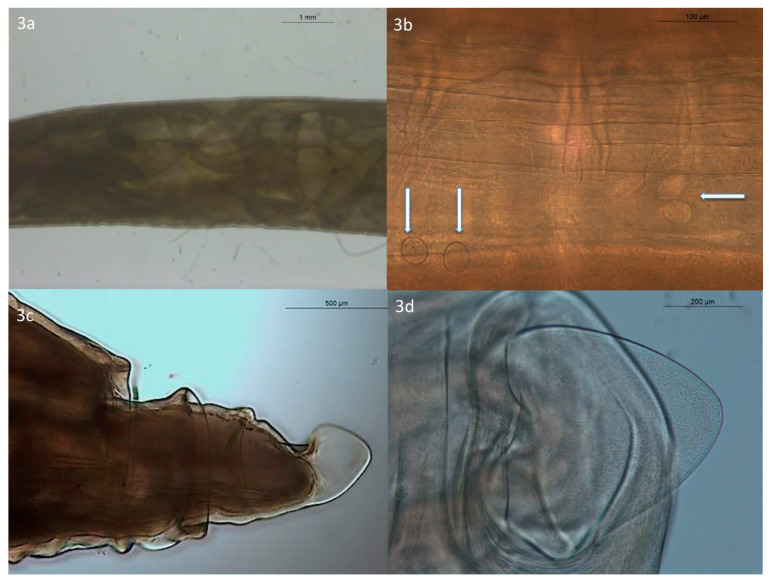
Light microscopy images of *A. caucasica* adult females. (**1a**) Buccal cavity. (**1b**) Cephalic collarette. (**1c**, **1d**) The two lateral pseudolabia. (**2a**) Esophagus. (**2b**) Thick finely striated from the mid-body. (**2c**) Pins. (**2d**) Vulva. (**3a**) Uteri. (**3b**) Eggs. (**3c**) Tail. (**3d**) Caudal appendix.

Comparative measurements of *A. caucasica* adult females from our study with *A. caucasica* (Linstow, 1902) and its synonymous species *P. mordens* (Lipper, 1908) [8] are detailed in Table 1. Two female complete specimens measuring 54.7 mm and 59.6 mm in length and 2.08 mm and 2.13 mm in width, respectively, were examined. The nematodes were characterized by the anterior end with a short buccal cavity (Figure 1(1a)) and by a cuticle reflecting over the lips to form a cephalic collarette (Figure 1(1b)) with two lateral pseudolabia undivided (Figure 1(1c,1d)). Nerve ring at 0.430 mm from the anterior end.

The esophagus consisting of two parts: muscular esophagus 0.75 mm long (Figure 1(2a)) and 0.287 mm wide, 0.79 mm long, and 0.268 mm wide, respectively. The esophagus total length was 5.52 mm and 4.82 mm, respectively in two samples.

In the mid-body, the cuticle was 0.70–0.122 mm thick and finely striated (Figure 1(2b)). The worms showed the presence of two small symmetrical pins in the front third of the body (Figure 1(2c)). Vulva 1.560 mm from anterior end (Figure 1(2d)). Presence of four uteri (Figure 1(3a)). Eggs were small 36–41 μm × 28–32 μm (Figure 1(3b)). Tail length was 1.084 mm (Figure 1(3c)). The caudal end showed the presence of a caudal appendix (Figure 1(3d)).

### 2.2. A. caucasica Eggs from Positive Feces 

**Figure 2:**
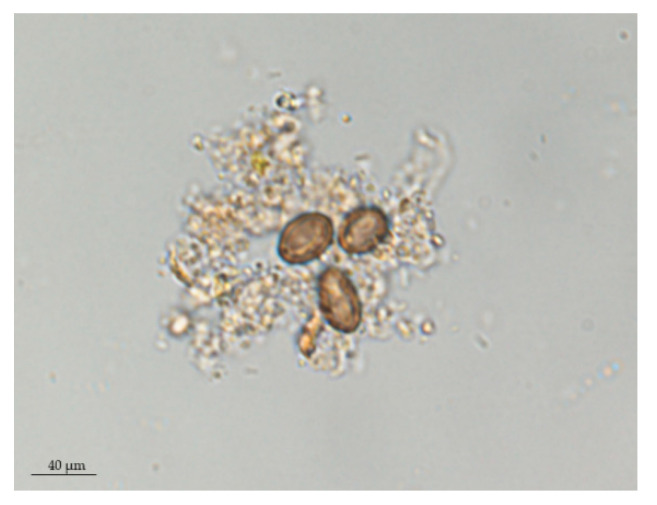
Coproscopy showing the *A. caucasica* eggs found in wild chimpanzee feces (formol-ether method, 100× magnification).

Eggs of *A. caucasica* were identified morphologically from two fecal samples taken from animals found infested with adult worms. The eggs were apparently identical to the micrograph of *A. caucasica* eggs reported elsewhere [7]. Eggs were embryonated and had a characteristic thick shell and hyaline coat (Figure 2). 

### 2.3. Molecular Characterization of Adult A. caucasica Worms 

First, nearly full-length DNA sequences of the 18S rRNA (AN: MN956824, MN956825), ITS2 (AN: MN956809, MN956810), *cox*1 (AN: MT231294, MT231295), 16S rRNA (AN: MN956826, MN956827), and 12S rRNA (AN: MN956811, MN956812) genes were obtained from adult worms of *A. caucasica.* The sequences of each gene were identical to each other. The BLAST analysis of 1140 bp of the 18S rRNA gene showed the highest query cover (100%) with eight sequences of *Physaloptera* sp. A nucleotide identity of 97.9% (1118/1142) was observed with *Physaloptera apivori* (EU004817) isolated from birds in Germany, followed by 97.89% (1116/1140) for both *Physaloptera* sp. (HM067978) isolated from long-tailed macaques (*Macaca fascicularis*) in China, and *Physaloptera turgida* (DQ503459) isolated from North American opossums (*Didelphis virginiana*) in Louisiana, USA. Finally, an identity ranging from 97.46% to 97.81% was observed within the five other sequences of *Physaloptera* species isolated from reptiles and mammals (EF180069, MG808040, JF934734, AY702703, and EF180065).

In contrast, the BLAST analysis of the partial (759 bp) *cox*1 nucleotide sequence showed the lowest values of query cover and identity with those of *Physaloptera* spp. from the GenBank database, having a greater sequence coverage (i.e., 98 to 100%) with those of Onchocercidae members than that observed for *Physaloptera* species (i.e., 83 to 88%). Among *Physaloptera*, the highest nucleotide identity values observed were 83.7% (529/632) with *Physaloptera* sp. (MH752202) isolated from brown anoles (*Anolis sagrei*) in the USA, 83.5% (530/635) with both *P. turgida* (KT894808) and *Turgida* sp. (KC130680) isolated from opossums (*Didelphis* spp.) in Brazil and Mexico, respectively, and 83.2% (558/671) with *P. amazonica* (MK309356) isolated from Gardner’s spiny rat (*Proechimys gardneri*) in Brazil, whilst lower identity values, ranging from 82.1% to 82.9%, were observed for the other five sequences of *Physaloptera* species (MH782844, KT894803, KT894804, KP981418, KT894805).

In contrast, the *Physaloptera cox*1 amino acid sequence appeared first among the top ten sequences of BLASTx [20]. *Abbreviata caucasica* COI sequence (protein id: QIP66136) showed an identity of 88.1% with *P. retusa* (AMX28288) isolated from golden tegu (*Tupinambis teguixin*) in Brazil, 87.7% with *P. mirandai* (AMX28289) isolated from brown four-eye opossums (*Metachirus nudicaudatus*) in Brazil with a coverage of 86% for both, 87.5% of identity and 98% of coverage with *P. rara* (QDF64304) isolated from dogs (*Canis lupus familiaris*), and 87.2% of identity and 86% of coverage with *Physaloptera* sp. (AMX28292), *P. bispiculata* (AMX28291), *P. amazonica* (QDX15779), and *P. hispida* (QCF40948). 

BLASTn analyses of 16S rRNA (416 bp) and 12S rRNA (573 bp) sequences identified the first 60 sequences that corresponded to those of *Filarioidea* and *Thelazidae* without any *Physaloptera*. Nucleotide identity of about 75% with a query coverage of more than 99% were observed among these Spiriruds. Finally, the BLASTn analysis of the partial (675 bp) sequence of the ITS2 showed an identity of 93.37% (67/71) and a coverage of 10% with the unique GenBank sequence of *Physaloptera alata* (AY702694) isolated from birds. 

**Figure 3:**
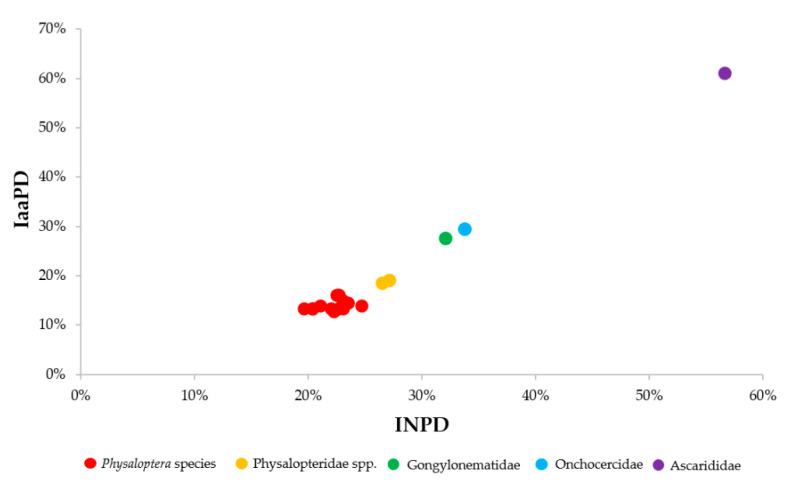
Scatter chart showing the interspecific pairwise distance between the COI sequence of *A. caucasica* and other nematodes based on both IaaPD and INPD. The INP distance was 0.31 (Std Err: 0.03) and 0.21 (Std Err: 0.06) between *A. caucasica* and *Heliconema longissimum* for THE 12S rRNA (GQ332423) and 16S rRNA (GQ332423) sequences, respectively. for the ITS2 sequences, the INP distance observed between *A. caucasica* and Filarioidea (XR 002251420, JQ316671, FM206482, DQ317666, DQ317657, and DQ317652), Spirocercidae (MH038181), Habronematidae (MH038181), and Gongylonematidae (LC026032, LC278392, and LC026029) members ranged from 0.51 (Std Err: 0.06) to 0.54 (Std Err: 0.06). No ITS2 sequences of Physalopteroidea superfamily were available.

The interspecific nucleotide pairwise (INP) distance of the 18S rRNA, *cox*1, 16S rRNA, 12S rRNA, and ITS2 of *A. caucasica* within *Physalopteridae* members are shown in Table S1. All sequences were well resolved in the chromatograms. The partial *cox*1 sequence was correctly aligned against the complete *cox*1 sequence (MH931178) of *P. rara* and no stop codon was observed in the translated amino-acid sequences, suggesting the absence of co-amplified numts. Furthermore, sequence alignment of COI with those of *Physaloptera* species showed nineteen amino-acid changes specific for *A. caucasica* (Figure S1). The interspecific nucleotide pairwise (INP) distance among the 645 bp of *cox*1 corroborated with the IaaP distance, among the corresponding 208 amino acid (Figure 3) and was substantially higher (ten times) between *A. caucasica* and *Physalopteridae* members in comparison with the 18S rRNA sequences. 

**Figure 4:**
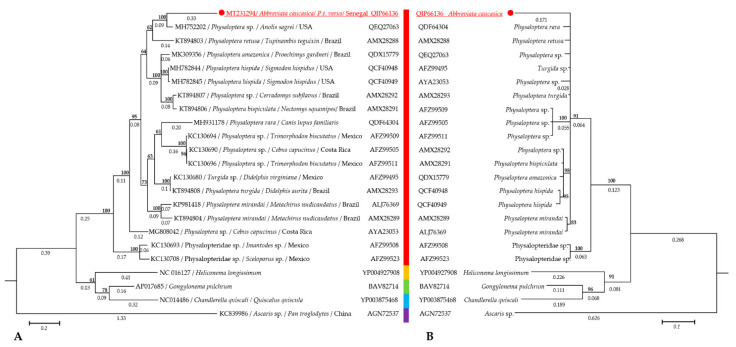
Phylogram generated from Bayesian inference. (**A**) Based on 651 bp from nucleotide sequences of the *cox*1 gene. (**B**) Based on 210 amino-acid from the COI-protein sequences. Numbers above and below branches are the display of nod statistics and branch length, respectively. Host, geographical location (when available), and GenBank accession numbers and protein-id are indicated. The identity of each taxa is color-coded according to the genus. Likelihood was −5448.2 and −1802.86 for nucleotide and protein inferences, respectively.

**Figure 5:**
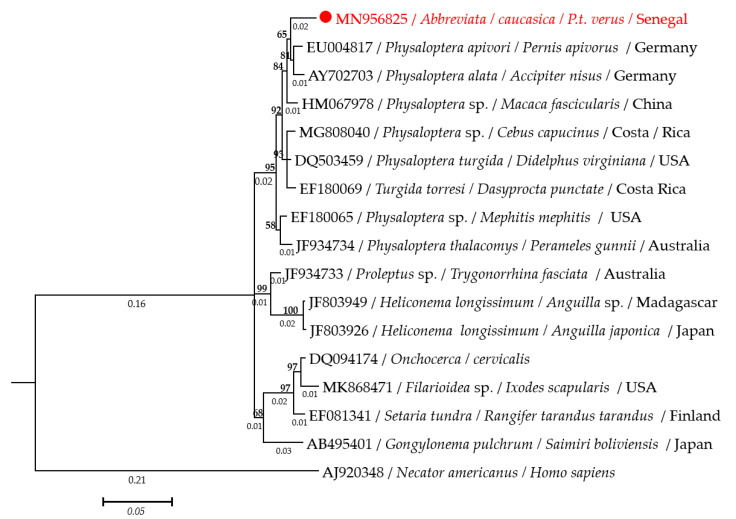
Phylogram generated from Bayesian inference, based on 1209 bp from 18S rRNA sequences. Numbers above and below branches are the display of nod statistics and branches length, respectively. Host, geographical location (when available), and GenBank accession numbers are indicated. Likelihood was −3466.3.

The Bayesian trees inferred from *cox*1, nucleotide, and protein sequences, and from 18S rRNA genes are shown in Figure 4A,B and Figure 5, respectively. All phylograms provide evidence that *A. caucasica* is an integral part of the genus *Physaloptera*. In particular, on the *cox*1 tree, *A. caucasica* clustered with *Physaloptera* sp. (MH752202) and *P. retusa* (KT894803) isolated respectively from *Anolis sagrei* in the USA and *Tupinambis teguixin* in Brazil (Figure 4A). Similarly, on the COI tree, *A. caucasica* clustered with *P. rara* (QDF64304), *P. retusa* (AMX28288), *Physaloptera* spp. (QEQ27063, AYA23053), *P. turgida* (AMX28293), and *Turgida* sp. (AFZ99495) (Figure 4B), while on the 18S rRNA tree, *A. caucasica* clustered together with *Physaloptera apivori* (EU004817) and *Physaloptera alata* (AY702703) isolated from birds in Germany (Figure 5).

**Figure 6:**
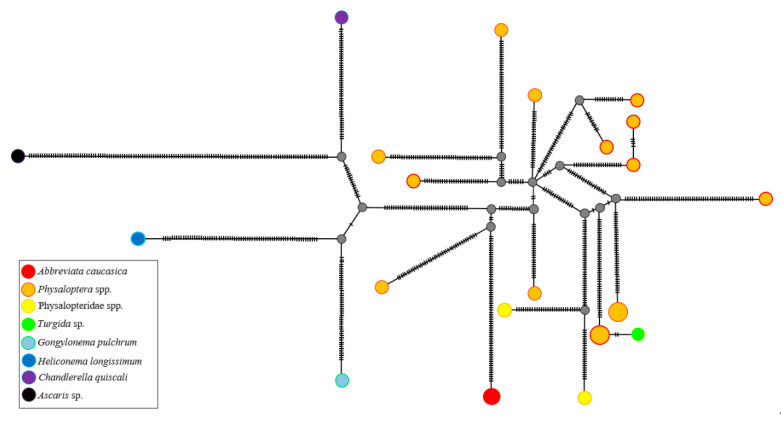
Templeton–Crandall–Sing (TCS) spanning network of the *cox*1 gene (651 bp) fragment. Colored and greyish circles correspond to a species genotype or hypothetical genotype, respectively. Model characteristics were: nucleotide diversity (pi = 1.282), number of segregating sites (361), number of parsimony-informative sites (242), Tajima’s D statistic (D = 30.3099), and *p* (“D >= 30.3099” = 0).

In addition, all *Physaloptera* and *A. caucasica* haplotypes shared a Euler circuit in the Templeton–Crandall–Sing (TCS) network tree for *cox*1 sequences. *Abbreviata caucasica* was connected by three-step branches to the Euler circuit, while all *Physaloptera* haplotypes were connected to the circuit by one to three-step branches (Figure 6). Hence, the TCS network analysis replicates the same results observed in the Bayesian inferences. 

### 2.4. Molecular Investigation of A. caucasica and Nematode Infestation from Biological Samples

The molecular tool developed in the present study was specific for the target DNA without any amplification from the negative controls. 

Results of the molecular screening for *A. caucasica* and nematode DNA are detailed in Table 2. Among the 48 fecal samples tested, 52.08% (*n* = 25) were positive for *A. caucasica*, while all environmental samples tested negative. In addition to the samples that tested positive for *A. caucasica*, the pan-Nematoda qPCR assay allowed for the detection of 29.17% (*n* = 14) of other fecal samples and 6.2% (*n* = 7, three soil samples from termite mounds and four termite specimens) of positive environmental samples. 

Fisher’s exact test showed that there were no significant effects of localities and fecal consistency on nematodes and *A. caucasica* prevalences (Table 2).

*Abbreviata caucasica cox*1 species-specific primers successfully amplified a partial sequence (504 bp) from 84% (21/25) of samples identified as positive by the qPCR targeting the 12S rRNA gene. There was no significant difference between both assays according to the McNemar test (*p* = 0.25). All sequences were identical to each other and showed 100% similarity to those from adult specimens amplified with pan-Nematoda primers. 

All sequences were deposited in the GenBank database under the following accession numbers: MT231296–MT231316.

### 2.5. The Analytical Sensitivity of A. caucasica 12S rRNA qPCR and Egg Counting 

The performance characteristics of the 12S rRNA qPCR are shown in Table S2 and Figure S2. The assay was species specific and was able to detect up to 1.13 × 10^−3^ eggs/g of positive fecal samples (i.e., corresponding to 1.13 × 10^−5^ eggs/5 μL of DNA). The qPCR efficiency was 101.8% with −3.28 and 28.68 as a Slope and Y-intercept values, respectively, allowing a perfect adjustment (R^2^ = 0.99).

Table 3 compares the *A. caucasica* eggs quantified by qPCR in terms of fecal consistency (fresh or degraded samples). Egg concentration in degraded feces (*n* = 4) was low (<1/g), but was higher (mean = 1.4 egg/g) in fresh feces (*n* = 21), while no effect of fecal consistency on egg concentration was observed (ANOVA, R2 = 0.032, Pr > F = 0.403).

## 3. Discussion

In this study, we report on the presence of *A. caucasica* (adults and eggs) in the feces of western chimpanzees from Senegal. Our data indicate that this population of chimpanzees is exposed to a high nematode infestation (81.3%), particularly *A. caucasica* (52.1%). This corroborates previous data from chimpanzees in southeastern Senegal, in which the reported nematode species-specific prevalence was between 0.78% to 31% where *Physaloptera* sp. was often the most prevalent species (13.26 to 31%) [12,14]. However, it was not specified whether these *Physaloptera* sp. were *A. caucasica* or author *Physaloptera* species. Perhaps the use of molecular assays, which were not applied in these studies, could offer a better species resolution. 

The adult worms were designated as *A. caucasica* after careful identification based on the morphological and morphometric features, which was strengthened by previous descriptions by Schulz, (1926), Ortlepp, (1926) and Brede and Burger, (1977) [8,9,21]. In addition to the morphological and morphometric features previously listed, we reported the width of the esophagus (0.268–0.287 mm) and that of the cuticle (0.70–0.122 mm), which may help in the future identification of *A. caucasica*. Morphologically, *Abbreviata* species are closely related to each other [22]. In 1945, Morgan described the utility of uterine morphology (number and mode of origin of the uteri in the female worm) in the taxonomic classification. He classified species from the genus *Abbreviata* (*n* = 27) into more than three classes with two (didelphys), four (tetra-delphys), or more than four (polydelphys) branches. Of those, three were associated with monkeys: *A. caucasica* (Linstow, 1902), *A. poicilomeira* (Sandground, 1936), and *A. multipapillata* (Kreis, 1940) [4]. Based on the uterine morphology, *A. poicilomeira* and *A. multipapillata* are listed in class 5–15 G (5–15 uteri with common trunk), and 9–13 H (9–13 uteri without common trunk), respectively. However, *A. caucasica* can be easily differentiated by the fact that it is in class 4-D (4 uteri with common trunk). 

The morphologic-based classification of Physalopteridae members (e.g., *Skrjabinoptera*, *Abbreviata*, and *Physaloptera*) exclude some morphometric measurements from the taxonomic characters such as the length of the esophagus, vulva position, and egg dimensions. These features seem to variate in the same species and are used only in exceptional cases such as *P. squamatae* (Harwood 1932), *S. chamaeleontis* (Gedoelst 1916), and *S. simplicidens* (Ortlepp 1922) [23]. As expected, our data confirmed the variability of these parameters within the *A. caucasica* (Table 1). This reduced the utility of some commonly used indexes (a, b, and c) in nematode taxonomy [24]. 

In addition to the important taxonomic characters highlighted by Fain and Vandepitte (1964) (e.g., morphological features of the anterior end posterior ands, the number of uterine branches), the two adult females measured in the present study exhibited morphometric features of body and egg size close to those of *P. mordens* (Lipper, 1908), a species synonymous with *A. caucasica* (Linstow, 1902), where the body size of the female is 41–100 × 1.8–2.8 mm with a small egg of 45–49 × 32–34 µm [7]. Eggs were also similar to those reported by Poinar et al., 1972, where the size is 35–40 × 25–35 µm [5]. However, *A. caucasica* (Linstow, 1902) has been described as having a small body size of 24.75–23.84 × 1.12–1.18 mm and larger eggs of 57–62 × 42–45 μm. In contrast, the measurements from the study of Fain and Vandepitte (1964) showed that the *A. caucasica* (syn. *P. mordens*) adult females had a big body size of 108–117 mm and larger eggs of 60–65 × 45–55 μm (Table 1). Furthermore, the same authors confirmed and described the inconsistency of some measurements within this species [7]. Traditional taxonomic keys are known to be inconclusive for the taxonomic classification of nematodes [25] and should be confirmed by molecular barcoding, which circumvents the limitations of classical morphology-based classification [26]. The question then arises of whether *A. caucasica* (Linstow, 1902) is the same specie as *P. mordens* (Lipper, 1908), as indicated by Ortlepp (1926) and Fain and Vandepitte (1964) using morphologic-based taxonomy [7,9]. To address this question, a molecular comparative characterization of the specimens from the studies of Schulz (1926) and Fain and Vandepitte (1964) [7,8] should be performed to confirm or refute the synonymy of these two species.

In our study, we expanded the genetic data available for *A. caucasica* with sequences of mitochondrial and nuclear DNA (i.e., *cox*1, 12S rRNA, 16S rRNA, 18S rRNA, and ITS2 genes), though the genetic characterization was based on *cox*1 and 18S rRNA genes, due to the limited data on other gene sequences of *Physalopterida* members in the GenBank database. 

The molecular analyses carried out in this study such as the phylogenetic comparisons of *cox*1 and 18S rRNA genes, the TCS network analysis of the *cox*1 gene, and the Bayesian inference of both *cox*1 and COI sequences confirmed that *A. caucasica* is monophyletic with *Physaloptera* species (Figure 4A,B and Figure 5). *cox*1 and 18S rRNA genes are widely used as markers for the molecular barcoding of nematodes [27] with *cox*1 sequences of relevance in resolving taxonomic relationships among nematode species [27,28]. This gene is described by an interspecific nucleotide pairwise distance (INPD) of 16% to 27.8% between nematodes species [29]. 

The description of new species from the genus *Physaloptera* as well as the recording of new hosts has quickly evolved over the last decade [30,31,32,33,34,35,36,37]. However, there is a lack of additional data on the epidemiology, life cycle, clinical signs, and description of larval stages in intermediate hosts, which impedes progress in the understanding of these parasites. This is also related to the limited diagnostic and monitoring methods, which has for long time been exclusively based on the identification of eggs in feces [1].

*Abbreviata caucasica* appears to be capable of living attached to the wall of the digestive tract between the esophagus and the small intestine in human and non-human primates [1,33]. However, clinical features of *A. caucasica* infestation in chimpanzees remain unknown at this time and further studies are needed to identify such features [6].

We developed a specific 12S qPCR-based assay for the detection of *A. caucasica* from biological samples and potential intermediate hosts, though the unique *Abbreviata* species DNA and target sequence from *A. caucasica* used to confirm the assay specificity may represent a limitation of the assay. In contrast, the newly *cox*1 *A. caucasica* specific PCR could be used to assess the identification of *A. caucasica* from hosts exposed to a wide range of nematode infestations. Since the PCR replicated the same result as the qPCR (*p* = 0.25), both tools were highly sensitive and specific in detecting *A. caucasica,* even the presence of coinfestations, avoiding the hard diagnosis based on egg identification. These tools can resolve problems related to the detection of larval stages from the intermediate and paratenic hosts and therefore avoid the sequencing identification by nematode generic primers. A detection limit as low as 1.13 × 10^−3^ eggs per gram of positive feces, regardless of consistency, solves the problems associated with conventional protocols requiring fresh equipment [38]. Data generated by qPCR showed a rate ranging from 0.2 to 1.4 eggs/g according to the fecal consistency, the best record being 113 egg/g. Appleton and Henzi (1993), reported the same results from baboons in Natal, South Africa, where egg output of *A. caucasica* ranged from 0.32 to 1.48 eggs/g with 215 eggs/g as the best record [39]. These observations highlight the usefulness of the qPCR quantification protocol we developed to evaluate the load of *A. caucasica* eggs. We therefore developed a 5S pan-Nematoda qPCR for the global exploration of nematode infestations from different biological samples. 

The absence of *A. caucasica* DNA from all environmental samples could be explained by the fact that they were not contaminated by the feces of infested hosts. However, despite the absence of *A. caucasica* DNA in the termite (*Isoptera* spp.) specimens that we tested, we cannot be sure if they are involved in the life cycle of *A. caucasica* or not. Termites (*Isoptera*) are the intermediate host of several nematodes such as *A. antarctica,* achanthocephalans (*Thorny-headed worms*), and *Heterakis gallinarum* [40,41,42]. 

Poinar and Quentin, (1972) experimentally demonstrated the ability of *Blatella germanica* and *Schistocerca gregaria* to develop the infective stage of *A. caucasica.* However, the life cycle of this nematode remains largely unknown. More than 28 paratenic and second intermediate hosts are also suspected [6]. However, we cannot be sure whether the environmental samples from species included in the diet of the chimpanzee population in our study, screened here, are not implemented in the life cycle of *A. caucasica* even in the absence of its DNA from all specimens. Termites are known to be the most prevalent arthropod in the chimpanzee diet [43].

## 4. Materials and Methods

### 4.1. Study Site and Study Subjects 

Samples were collected at the Dindefelo Community Natural Reserve, located in the Kedougou region, southeastern Senegal, about 35 km from the town of Kedougou. The vegetation of the reserve is a sudano-guinean savanna woodland [44], one of the driest and more open habitats occupied by the species [45]. All chimpanzees live in multi-female/multi-male communities composed of flexible groups that fission and fuse [46]. At the time of data collection, some individuals were semi-habituated to observers, but the rest remained unhabituated and thus the exact community composition and size were unknown. Although the total home range of Dindefelo chimpanzees was not known, conspecifics living in savanna woodland habitats have extremely large home ranges (e.g., >85 km^2^, [47]). Based on size, the fecal samples analyzed in this study were assumed to belong to adults.

### 4.2. Fecal, Worms, and Environmental Samples 

**Figure 7:**
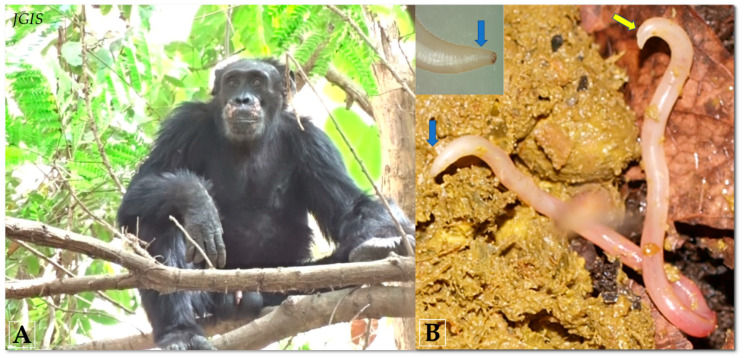
Adult of A. caucasica nematode found in the fecal matter of wild chimpanzee from the Dindefelo Community Natural Reserve, Senegal. (**A**) A chimpanzee (*Pan troglodytes verus*) in its natural habitat. (**B**) Adult female of *A. caucasica* looks like *Ascaris* with smooth and elastic body, strangled head, circular mouth (blue arrow), and incurved tail (yellow arrow).

Two expeditions to the Dindefelo Community Natural Reserve in Senegal were undertaken in order to collect the samples. During the first trip (August 2016), 49 fecal samples of the western chimpanzee (*Pan troglodytes verus*) (Figure 7A) were collected from three localities in the reserve: Locality 1, three decomposing “degraded” fecal samples (12.382539, −12.287977); Locality 2, six degraded fecal samples (12.381437, −12.290776); and Locality 3, thirty-eight fresh fecal samples (12.379919, −12.296830). The fecal samples were collected and stored at −80 °C. Two adult worms were recovered from two fresh feces in the field and stored in 70% ethanol. In shape and general appearance, these worms resembling *Ascaris* to the naked eye (Figure 7B). All samples were transported to our laboratory at the Institut Hospitalo-Universitaire (IHU) Méditerranée Infection for further examination, and the adult worms were sent to the parasitology laboratory of the Department of Veterinary Medicine (University of Bari, Italy), where they were subjected to morphological identification. During the second expedition (August 2019), we targeted the potential contamination of these parasitic nematodes for the chimpanzees (e.g., the possible intermediate hosts that chimpanzees could eat or their water sources). A total of 113 environmental samples including the main species from the chimpanzee’s diet were collected in the vicinity of chimpanzee sleeping sites and other areas frequented by the apes. These included 47 termites, 42 soil samples from termite mounds, 21 plant species, one sample from a water source used by the chimpanzees, a centipede, and one maggot. All samples were preserved in 70% ethanol and were transported to our laboratory for analysis.

### 4.3. Morphological Analysis of A. caucasica Adult Worms

The female worms were processed for morphometric analysis. The body of the nematodes were measured and then cut into three pieces. The central part was subjected to DNA extraction for molecular identification. The cephalic and caudal ends of the worms were fixed and stained in lactophenol solution to observe anatomical structures. Digital images and measurements were made with an optic microscope Leica^®^ DM LB2 with differential interference contrast. The software Leica^®^ LASAF 4.1 was used for the image analysis process including the measuring of nematodes, which are provided in micrometers. The identification was carried out following the description made by Schulz, (1926), Ortlepp, (1926) and Brede and Burger (1977) [8,9,21].

The observation of structures in the cephalic region, the stout size of the nematode, a thick cuticle finely striated, a large cephalic collarette, the total length, and the distance from the anterior end to the vulva all confirmed the identification of this helminth as *A. caucasica*.

### 4.4. Identification of A. caucasica Eggs from Positive Feces

The exploration of *A. caucasica* eggs was investigated from two fecal samples from which the adult worms were collected. A formol-ether sedimentation method of fecal concentration was used [48]. Egg identification was carried out according to the key of Fain and Vandepitte (1964) [7], while the differential diagnosis was performed as described elsewhere [49].

### 4.5. DNA Extraction

Genomic DNA was extracted from 200 mg of fecal samples, adult worms of *A. caucasica*, and environmental specimens using the QIAGEN DNA tissue kit (QIAGEN, Hilden, Germany) following the manufacturer’s recommendations. The extraction was performed after two lysis steps: (i) mechanical lyses performed on a FastPrep-24™ 5G homogenizer using high speed stirring for 40 s in the presence of the powder glass, and (ii) enzymatic digestion using the proteinase K in the appropriate buffer (QIAGEN, Hilden, Germany) for 12 h at 56 °C. The extracted DNA was then eluted in a total volume of 100 µL and stored at −20 °C. 

### 4.6. Molecular Characterization of Adult Worms 

#### 4.6.1. Development of PCR Primer Sets

The primer sets used in this study are listed in Table 4. First, sequences of the cytochrome *c* oxidase I (*cox*1), 16S rRNA, 12S rRNA, and the internal transcribed spacer 2 (ITS2) genes were used to design primer sets targeting nematodes. For each PCR system, a fasta file was constructed from nematode sequences retrieved from the GenBank database. Sequences were aligned using BioEdit v7.0.5.3 software [50]. The highly conserved areas were submitted in Primer3 software v. 0.4.0 [51]. PCRs standardization was performed as described elsewhere [52]. Primers designed are reported in Table 4.

In addition, primers Fwd.18S.631 and Rwd.18S.1825r, recently designed to amplify a partial fragment of the 18S rRNA gene of nematodes, were also used in this study (Table 4) [53]. These genes were chosen in order to compare the relatedness with *Physaloptera* and parasitic nematodes available in the GenBank database.

#### 4.6.2. Polymerase Chain Reaction, Sequencing and Phylogenetic Analysis 

All PCR reactions were carried out in a total volume of 50 µL, consisting of 25 µL of AmpliTaq Gold master mix (Thermo Fisher Scientific), 18 µL of ultra-purified water DNAse-RNAse free, 1 µL of each primer, and 5 µL of genomic DNA. PCR reactions with all systems were run using the following protocol: incubation step at 95 °C for 15 min, 40 cycles of 1 min at 95 °C, 30 s for the annealing at a different melting temperature for each PCR assay, and elongation for 45 s to 1 min and 30 s (Table 4) at 72 °C with a final extension for 5 min at 72 °C. PCR reactions were performed in a Peltier PTC-200 model thermal cycler (MJ Research Inc., Watertown, MA, USA).

The amplicons obtained from each gene examined were purified using the filter plate Millipore NucleoFast 96 PCR kit following the manufacturer’s recommendations (Macherey Nagel, Düren, Germany). Purified DNAs were subjected to the second amplification using the BigDye™ Terminator v3.1 Cycle Sequencing Kit (Applied Biosystems, Foster City, CA, USA). Then, BigDye PCR products were purified on the Sephadex G-50 Superfine gel filtration resin prior to sequencing on the ABI Prism 3130XL.

First, all nucleotide sequences were assembled and edited by ChromasPro 2.0.0. The absence of co-amplification of nuclear mitochondrial genes (numts) was verified for the *cox*1 DNA sequences, wherein the alignment was performed with the complete sequence of *cox*1 DNA from the *P. rara* mitochondrion sequence (MH931178) using the ClustalW application within Bioedit v.7.2.5. [50]. In addition, the visual verification of sequence chromatograms ambiguities, indels and stop codons of the translated sequences was performed using Chromas Pro 2.0.0 software as recommended [54]. Sequences amplified from the *cox*1, 16S, 12S, 18S rRNA, and ITS2 genes were subjected separately to a preliminary analysis using the Basic Local Alignment Search Tool (BLAST) [55]. 

The closely related sequences of *Physaloptera* and nematode species retrieved from the GenBank database were included in the study and a fasta file was constructed for each gene and then subjected to the alignment. In addition, alignment of nematode COI protein sequences was also performed. All alignments were conducted using the ClustalW application within Bioedit v.7.2.5. [50]. The conservation of amino acids between the COI sequences of *A. caucasica* relative to the sequences of *Physaloptera* was visualized on CLC Sequence Viewer 7 (CLC Bio Qiagen, Aarhus, Denmark).

From the alignment of each gene examined, the interspecific nucleotide pairwise distance (INPD) was evaluated to estimate the genetic divergence between all species included. Furthermore, the interspecific amino acid pairwise distance (IaaPD) was reproduced for COI-protein sequence alignment. standard errors were obtained by a bootstrap procedure with 1000 replicates using the maximum composite likelihood model [56] and Poisson correction model [57] for nucleotide and protein sequence alignments, respectively. Evolutionary analyses were inferred on MEGA6 software [58].

DNA sequences of *Necator americanus* (AJ920348) and *Ascaris* sp. (KC839986) were chosen as out-groups for18S rRNA and *cox*1, respectively, according to the fast-minimum evolution tree on BLAST [55]. The corresponding COI protein sequence of *Ascaris* sp. (AGN72537) was maintained as an out-group for the COI-protein alignment. The best model parameters with the lowest score were selected to generate phylogenetic trees of aligned 18S and *cox*1 sequences as well as COI protein sequences by running the MrBayes algorithm on each model using Topaliv2.5 software [59]. The Bayesian phylogenetic tree [60] was inferred for nucleotide sequence alignments using the K80 (+G) [61] and GTR (+G, +I) [62] models, respectively, while the Bayesian phylogenetic tree was inferred on the COI protein sequence alignment using Mtmam (+G) [63]. All phylograms were generated with five runs for 1,100,000 generations, 25% of burn-in length, and 1000 for sample frequencies.

In order to resolve the haplotype variations of *Physaloptera* species and *A. caucasica*, the Fasta file of the *cox*1 sequences was converted to the Nexus format using an online converter [64]. During the second time, a Templeton–Crandall–Sing (TCS) network phylogram [65] was inferred with a 95% connection limit and drawn with 1000 iterations using the PopArt software [66]. 

### 4.7. TaqMan qPCR for Nematoda Parasites Detection 

The 5S rRNA gene was selected for the development of a TaqMan qPCR as an exploratory tool targeting nematode parasites. This choice was based on its conservation among nematodes species [67] and its tandem repetition of 110 times, which improves the assay sensitivity [68]. The partial sequences (XR002251414, JX489168, HM641830, M27961, JX117890, LS997562, U32120, AP018154, LK928622, X16226) representing nematode members from *Spirurina* and *Rhabditida* clades were aligned against the 5S gene of some plathelminth worms (*Schistosoma mansoni*: XR001974633, *Spirometra erinaceieuropaei*: LN313518, *Hymenolepis microstoma*: LR215994) and vertebrate hosts (human: AC275639, dog: XR003137316, chimpanzee: XR002941379, horse: XR002802613). Primers: qNem.5S.1f 5′-ACCACGTTGAAAGCACGMC-3′; qNem.5S.110r 5′-TGTCTACAACACCTSGRATTCC-3′; Eurogentec (Liège, Belgium), and a TaqMan probe qNem.5S.38p 6-FAM-5′-AGTTAAGCAACGTTGGGCC-3′-TAMRA; Applied Biosystems^TM^, were chosen from the highly conserved region specific for nematodes. 

### 4.8. Quantitative TaqMan Real-Time PCR (qPCR) for A. caucasica Detection 

Sequences of the 12S rRNA gene amplified from the adult worms of *A. caucasica* were aligned with the closely related sequences of nematodes available in the GenBank using Bioedit v.7.2.5. [50]. The specific regions for 12S rRNA of *A. caucasica* were submitted in Primer3 software v. 0.4.0 [51] in order to design the following primers Phy.12S.f.204:5′-GAATTGGATTAGTACCCAAGTAAGTG-3′/Phy.12S.r.305: 5′-TGTTCCAAAAATCTTTCTAAGATCAG-3′ (Eurogentec, Liège, Belgium) and TaqMan probe: Phy.12S.242p. 6VIC-GCGGGAGTAAAGTTAAGTTTAAACC-TAMRA) (Applied Biosystems^TM^), allowing the amplification of a fragment of 101 bp. 

Both qPCRs were tested in silico within the DNA databases of metazoans (taxid: 33208), vertebrates (taxid: 7742), bacteria (taxid: 2), *Canidae* (taxid: 9608), Felidae (taxid: 9682), and humans (taxid: 9605). This experiment was performed for both combinations of forward-reward and probe-reverse of each qPCR using Primer-BLAST [69]. Subsequently, the specificity was also validated in vitro against the genomic DNA extracted from adult worms of *A. caucasica* and DNA database including several nematodes, arthropods, laboratory-maintained colonies, hemopathogens as well as human, monkey, donkey, horse, cattle, mouse, and dog as described elsewhere [52]. 

All qPCR reactions included 5 μL of DNA template, 10 μL of Master Mix Roche (Eurogentec), and 3 µL of ultra-purified water DNAse-RNAse free. Concentration of each primer, UDG, and each probe was 0.5 µL. The TaqMan reaction of both systems was run using the same cycling conditions. This included two hold steps at 50 °C and 95 °C for 2 and 15 min, respectively, followed by 40 cycles of two steps each (95 °C for 30 s and 60 °C for 30 s). The qPCR reaction was performed in a CFX96 Real-Time system (Bio-Rad Laboratories, Foster City, CA, USA) after activating the appropriate dye readers for each qPCR system.

A protocol for the quantification of eggs has been established to assess the analytical sensitivity of qPCR in the detection of fecal infestation. A 10-fold serial dilution of DNA extracted from 200 mg of fecal matter containing 113 eggs (Figure 2) per gram (i.e., 22.6 eggs/100 µL of eluted DNA and 1.13 eggs/5 µL of qPCR reaction). Standard curves and derived parameters (PCR efficiency, Slope, Y-intercept, and correlation coefficient) were generated using CFX Manager Software Version 3 [70]. 

The molecular approaches described above were used to screen the presence of *A. caucasica* and other nematodes in chimpanzee fecal and environmental samples collected in a chimpanzee dormitory.

### 4.9. Conventional PCR Specific for A. caucasica

The use of universal pan-Nematoda primers does not allow for the identification of species-specific DNA sequences due to a non-specific amplification in co-infestations. A specific *cox*1-based PCR was developed in order to complete the identification of *A. caucasica* from fecal samples. The specific region for *A. caucasica* was analyzed for the design of the primers COI.51f and COI.601r, targeting 550 bp of the *cox*1 gene (Table 1). *A. caucasica cox*1 partial sequences herein amplified by the pan-Nematoda primers from the adult worms were aligned with *Heliconema longissimum* (AN: GQ332423) and *Gongylonema pulchrum* (AN: AP017685), representative members of *Physalopteroidea* and *Gongylonematidae*, respectively. 

### 4.10. Molecular Survey of A. caucasica and Nematode Infestations in a Chimpanzee Population and the Environmental Samples

DNA from fecal samples of chimpanzee (*n* = 48) and environmental samples (*n* = 113) were screened for the DNA of *A. caucasica* and nematode using the 12S rRNA *A. caucasica* and the 5S rRNA pan-Nematoda qPCR assays, respectively. Positive samples for *A. caucasica* were also subjected to amplification and sequencing using the *cox*1 *A. caucasica*-specific primers. 

### 4.11. Statistical Analysis 

XLSTAT Addinsoft version 4.1 (XLSTAT 2019: Data Analysis and Statistical Solution for Microsoft Excel, Paris, France) was used for the statistical analysis. Results of qPCRs analysis were used to set a database using the Microsoft Excel^®^ program (Microsoft Corp., Redmond, Washington, USA). The effect of localities and fecal consistency on the infestation rates were tested using the Khi2 test or exact Fisher test. One-way analysis of variance (ANOVA) was performed to compare the predicted eggs from fresh and degraded feces. Negative samples and those with a studentized residual higher than 2.9 were removed before discarding the ANOVA test. McNemar’s test was used to compare the detection accuracy of the qPCR and conventional PCR of *A. caucasica* from the chimpanzee samples. Significance level was considered at alpha ≤ 0.05 for all analyses. 

## 5. Conclusions

*A. caucasica* measurements indicated the inconsistencies of certain indexes such as index a, b, and c (Table 1) within this nematode, while it remains distinguishable from other *Physaloptera* species by the morphological features of the anterior and posterior ends as well as the presence of four uteri with a common trunk. However, the phylogenetic analyses showed that *A. caucasica* are clustered together with other monophyletic species of the *Physaloptera* genus. In the absence of strong morphological and epidemiological data, the species of *Abbreviata* may be re-classified as *Physaloptera* and a revision of the genus is needed. We developed specific and reliable molecular tools for the detection and egg quantification of *A. caucasica* from fecal samples. The tests can ultimately help to identify possible intermediates as well as paratenic hosts involved in the life cycle of *A. caucasica*. We therefore investigated its prevalence in a chimpanzee population from Senegal. Further studies are needed to clarify the epidemiology, circulation, life cycle, and possible pathological effects of *A. caucasica*, and the role of paratenic hosts or arthropods as intermediate hosts.

## Figures and Tables

**Table 1:** Comparative measurement (in mm unless specified) of adult female of *A. caucasica* from our study with *A. caucasica* (Linstow, 1902) and its synonymous species *P. mordens* (Lipper, 1908) according to Fain and Vandepitte (1964) [7], Linstow (1902) and Schulz (1926) [8].

Measures		This Study		Measurement of Fain and Vandepitte (1964)		Measurement of Linstow, 1902	Measurement of Schulz (1926)			
		*A. caucasica*		*A. caucasica (Syn. P. mordens)*		*A. caucasica: Type Species*	*A. caucasica: Type Species*		*P. Mordens: Type Species*	
		Chimpanzee (Senegal)		Men (Congo)		Men (caucasia)	Men (caucasia)		Men (Uganda)	
Organ	Segment	CHS 11	CHS 31	N1	N1	Adult Female	N1	N2	N1	N2
**Body**	Length	54.7	59.6	108	117	27	24.75	23.84	41	100
	Width	2.08	2.13	2	3	1.14	1.18	1.12	1.8	2.8
	Index a	26.3	28	54	39	23.68	20.97	21.29	22.78	35.71
**Nerve ring**	From the anterior end	0.43	-	0.7	0.78	-	0.454	0.454	-	-
**Esophagus (e)**	Total length	5.52	4.82	9	11	-	3.5	3.72	-	-
	Length muscolar (e)	0.79	0.75	0.6	0.6	-	0.43	0.35	-	-
	Width	0.268	0.287			-	-	-	-	-
	Index b	9.91	12.37	12	10.63	-	7	6.4	6.2	6.2
**Cuticle**	Width	0.92–0.102	0.70–0.122	-	-	-	-	-	-	-
**Vulva**	From the anterior end	1.56	-	21	23	-	3.50	4.62	-	-
**Eggs (μm)**		37–41 × 28–32	36–39 × 28–31	60–65 × 45–55		57 × 39	57–62 × 42–45		45–49 × 32–34	
**Tail**	Tail	1.084	-	1.3	1.4	0.51	0.578	0.532	-	-
	Index c	50.46	-	80	83	53	43	45	70	90

CHS 11 and CHS31: Code sample of specimens from the present study. N1, N2: Code samples of specimens from the study of Schulz (1926). Index a, b, and c are the ratio of body length to body width, esophagus length and tail length, respectively.

**Table 2:** *Abbreviata caucasica* and nematode infestations regarding origin and fecal consistency.

	Tested Samples	Infestation Rate (%) by qPCRs	
		A. caucasica	Nematodes
**Localities**			
**Locality 1**	3	0	33.3
**Locality 2**	6	66.7	100
**Locality 3**	39	53.8	82.1
**Total**	48	56.3	81.3
**Comparison by localities**	Fisher test (p)	0.148	0.052
**Fecal consistency**			
**Fresh**	38	40.0	70.0
**Degraded**	10	55.3	84.2
**Fisher test (p)**	//	0.49	0.30

**Table 3:** *Abbreviata caucasica* egg output from the positive samples.

Fecal Consistency	Number of Positive Samples	Quantification (Means Eggs/g) from Positive Samples	
**Degraded**	4	0.2	
**Fresh**	21 *	1.4	
**Statistics**	One-way ANOVA	R2	0.032
		Pr > F	0.403

*: one sample with an abnormal residual was removed before statistical analysis. Degraded: decomposing fecal samples.

**Table 4:** Primer sets used for the molecular characterization of *A. caucasica.*

Primer Name	Sequences 5′-3′	Target Gene	Size (bp)	Melting Tm	Elongation Time	Specificity	Ref.
**Fwd-ITS-793**	TCGATGAAGAACGCAGCTA	ITS2	420–750	57 °C	1’	Pan-Nematoda	This study
**Rwd-ITS-1495**	AGTTTCTTTTCCTCCGCTTAGTT						
**Fwd-12S-Nem-1**	AAGTTTGATTTTGGTTTTGGTTG	12S	680	58 °C	1’		
**Rwd-12S-Nem-681**	CCATTGACGGATGGTTTGTA						
**Fwd-16S-Nem-488**	GCAGCCTTAGCGTGATGG	16S	430	58 °C	1’		
**Rwd-16S-Nem-918**	TAAACCGCTCTGTCTCACGA						
**dg.Fwd.COI.Nem.257**	TTGGKGGTTTTGGWAATTGG	*Cox* 1	1069	52 °C	1’30”		
**dg.Rwd.COI.Nem.1325**	CCAGCAAAATGCAWAGGAAAA						
**Fwd.18S.631**	TCGTCATTGCTGCGGTTAAA	18S	1127–1155	54 °C	1’30”	Pan-Nematoda	[53]
**Rwd.18S.1825**	GGTTCAAGCCACTGCGATTAA						
**Fwd.Abbrev.COI.51f**	TGATCAGGGTTGGGAGCTT	*Cox* 1	550	53 °C	1’	*A. caucasica*	This study
**Rwd.Abbrev.COI.601r**	AAAAAGAACAATTAAAATTACGATCC						

## Supplementary Materials

The following are available online at https://www.mdpi.com/2076-0817/9/7/517/s1. Figure S1. *Abbreviata caucasica* partial COI-protein sequences alignment against *Physaloptera* species. The sequences of *A. caucasica* (selected box) obtained from adult worms were aligned against Physaloptera sequences available in the GenBank database. Residues were matched as dots. Conserved areas are indicated in blue, while the intensity of mutations is indicated by a foreground color (red to black). Figure S2. Quantification protocol of the 12S *A. caucasica*-specific qPCR. (**A**) Determination of detection limits and efficiency (eggs/g of fecal matter). (**B**) Standard curves generated from a serial 10-fold dilution of DNA.
