# Supplementary material for: Zoonotic Abbreviata caucasica in Wild Chimpanzees (Pan troglodytes verus) from Senegal
Source: Pathogens. 2020 Jun 27;9(7):517. doi: 10.3390/pathogens9070517 (PMC7400140; doi:10.3390/pathogens9070517)
Supplement: Supplementary file 1 [file pathogens-09-00517-s001.zip › Table S2.docx]

**Table S2:** Sensitivity and assay performance characteristics of 12S qPCR-based system in detecting *A. caucasica* eggs

| Starting Quantity (SQ) eggs/g | SQ per qPCR reaction from eggs/5µl | Cq Mean | E-RFU | SCRS |
| --- | --- | --- | --- | --- |
| 1.13× 10^+2^ | 1.13× 10^0^ | 21.2 | 1355 | (E= 101.8%) (S=-3.281)  (Y.int=28.677)  (R^2^=0.995) |
| 1.13× 10^+1^ | 1.13× 10^-1^ | 23.8 | 1575 |  |
| 1.13× 10^0^ | 1.13× 10^-2^ | 27,0 | 1395 |  |
| 1.13× 10^-1^ | 1.13× 10^-3^ | 30.5 | 1097 |  |
| 1.13× 10^-2^ | 1.13× 10^-4^ | 33.4 | 679 |  |
| 1.13× 10^-3^ | 1.13× 10^-5^ | 37.7 | 224 |  |
| 1.13× 10^-4^ | 1.13× 10^-6^ | 0.0 | 41 |  |
| 1.13× 10^-5^ | 1.13× 10^-7^ | 0.0 | 35.9 |  |
| 1.13× 10^-6^ | 1.13× 10^-8^ | 0.0 | 33 |  |
| 1.13× 10^-7^ | 1.13× 10^-9^ | 0.0 | 28.1 |  |
| Cut Off Value | | 38.0 | 41.3 |  |
| Negative Control | | N/A | 30.5 |  |

**Cq**: cycle quantification value; **N/A**: No amplification, **E-RFU**: End of relative fluorescence unit, **SCRS**: Standard Curve Results Spreadsheet, **E**: Efficiency, **S**: Slope, **Y.int:** Y-intercept.
