# Supplementary figures and images for: Zoonotic Abbreviata caucasica in Wild Chimpanzees (Pan troglodytes verus) from Senegal
Source: Pathogens. 2020 Jun 27;9(7):517. doi: 10.3390/pathogens9070517 (PMC7400140; doi:10.3390/pathogens9070517)

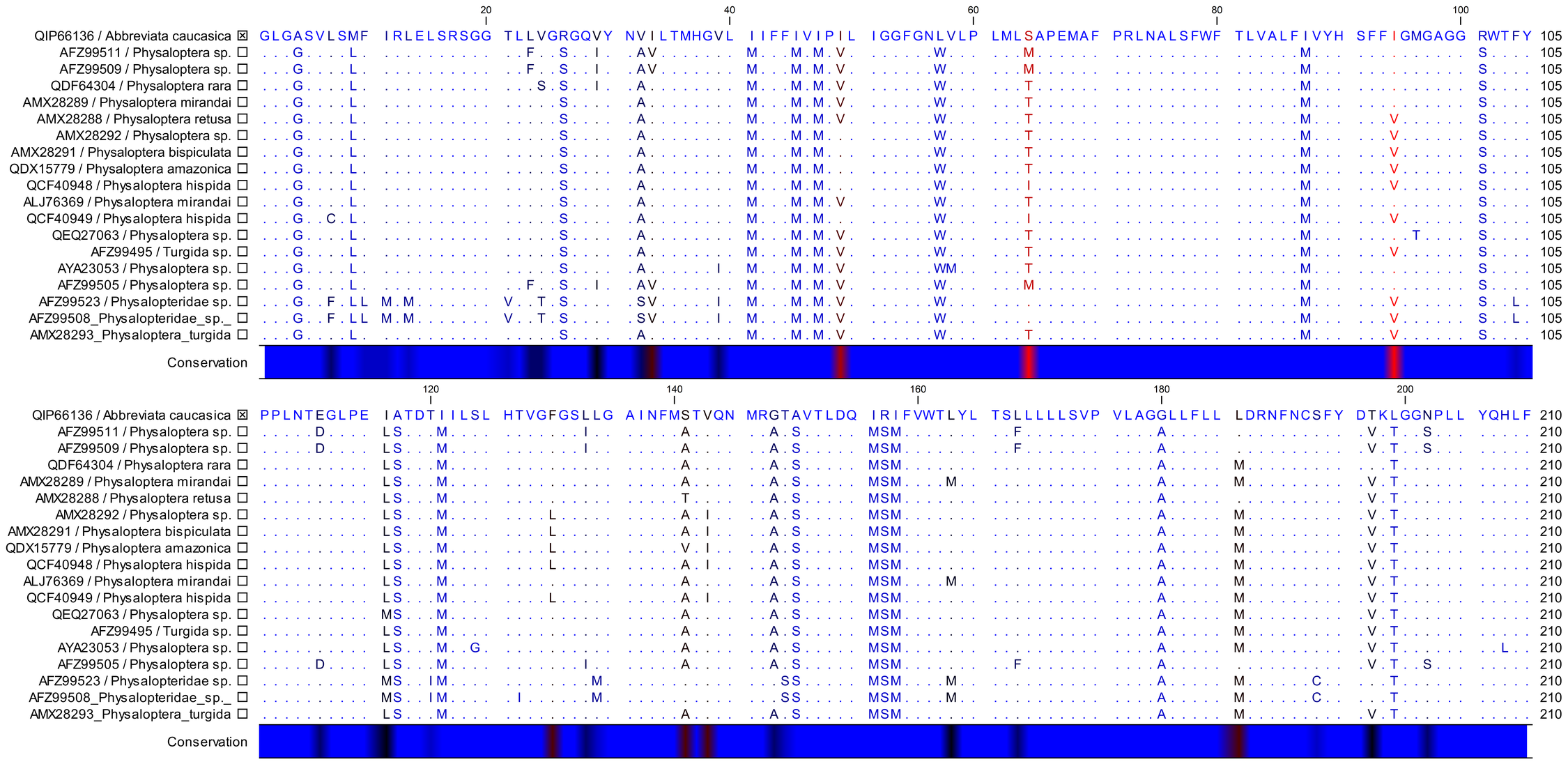

Supplement: Supplementary file 1 [file pathogens-09-00517-s001.zip › Figure S1.tif]

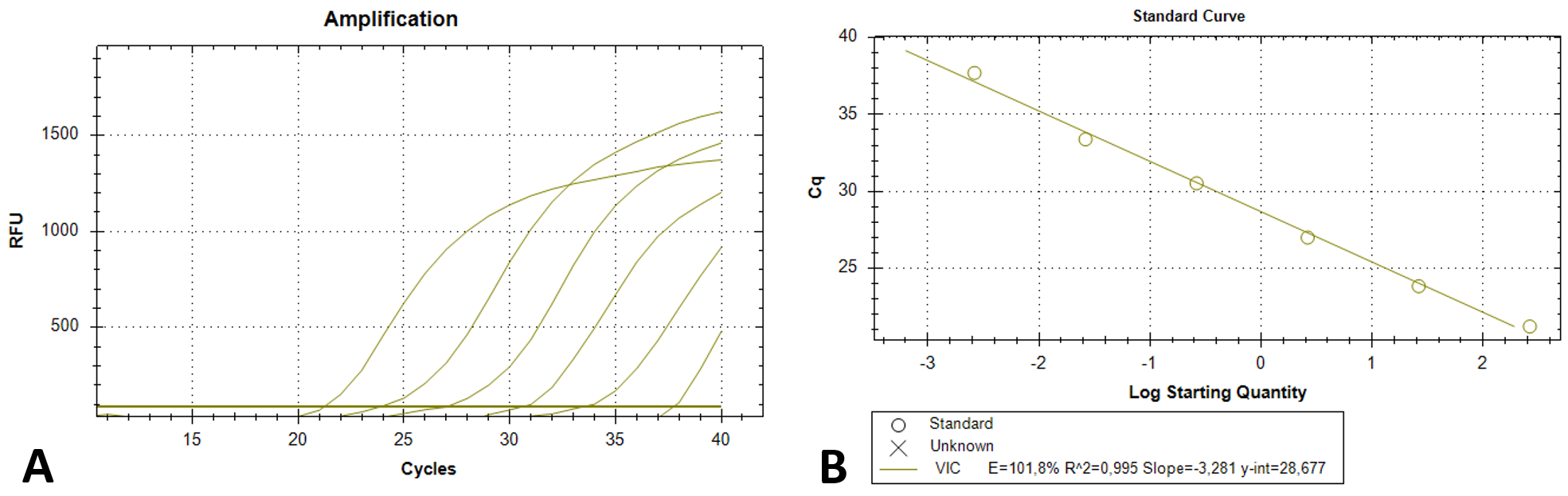

Supplement: Supplementary file 1 [file pathogens-09-00517-s001.zip › Figure S2.tif]
